# Poorly Differentiated Thyroid Carcinoma Arising in Struma Ovarii

**DOI:** 10.1155/2015/826978

**Published:** 2015-06-21

**Authors:** Surapan Khunamornpong, Jongkolnee Settakorn, Kornkanok Sukpan, Prapaporn Suprasert, Sumalee Siriaunkgul

**Affiliations:** ^1^Department of Pathology, Faculty of Medicine, Chiang Mai University, Chiang Mai 50200, Thailand; ^2^Department of Obstetrics and Gynecology, Faculty of Medicine, Chiang Mai University, Chiang Mai 50200, Thailand

## Abstract

Struma ovarii is an uncommon type of ovarian mature teratoma with a predominant thyroid component. The morphological spectrum of the thyroid tissue ranges from that of normal thyroid to proliferative adenoma-like lesions and thyroid-type carcinomas (malignant transformation). The histologic features of ovarian strumal lesions sometimes cause diagnostic problems due to the confusion with other types of ovarian neoplasms and the difficulty in the prediction of their clinical behavior. We report an extremely rare case of poorly differentiated thyroid carcinoma arising in struma ovarii. A 22-year-old woman presented with a 15 cm right ovarian mass. The tumor showed a predominantly tubular pattern which raised a differential diagnosis between endometrioid adenocarcinoma and Sertoli cell tumor. A review of the gross specimen with additional tissue sampling helped identify the teratomatous and strumal nature, with a support by immunohistochemical staining. Despite FIGO stage IA by optimal staging procedure and the absence of identifiable lymphovascular invasion, the patient developed pulmonary metastasis 15 months after surgery and died from the progression of the disease 7 years after the diagnosis. This case emphasizes the importance of macroscopic examination of the specimen and the awareness of this uncommon tumor in the differential diagnosis of ovarian neoplasms.

## 1. Introduction

Struma ovarii is an uncommon type of ovarian mature teratoma which is composed predominantly (i.e., > 50% of tumor component) or purely of thyroid tissue [1]. The morphological spectrum of the thyroid component ranges from that similar to normal thyroid, cellular or “proliferative” lesions with adenoma-like or hyperplastic features, to thyroid-type carcinomas similar to those of the thyroid (malignant transformation) [2, 3]. The histologic features of the proliferative and malignant spectrum in struma ovarii sometimes cause diagnostic confusion with other types of ovarian neoplasm, particularly sex cord-stromal and epithelial tumors [4, 5].

A malignant transformation of ovarian struma is very rare, with an estimated annual incidence of less than 1 in 10,000,000 women-years [2]. Although it is sometimes referred to as “malignant struma ovarii,” this term represents a heterogenous group of tumors. A better diagnosis should include a specific type of thyroid carcinoma that arises in a strumal background as this would provide meaningful prognostic information [6, 7]. Malignancy arising in struma ovarii can pose diagnostic problems for pathologists in the identification of the strumal nature and also in the determination of tumor behavior [2]. A correct diagnosis is very important due to major differences in the therapeutic approaches among tumors which are included in the differential diagnoses.

This report describes an extremely rare case of poorly differentiated thyroid carcinoma arising in struma ovarii. This case emphasizes the importance of macroscopic examination of the specimen and the awareness of this uncommon tumor in the differential diagnosis of ovarian neoplasms.

## 2. Case Report

### 2.1. Clinical History

A 22-year-old woman presented with increased abdominal girth which she had experienced for 5 months. Physical examination and pelvic ultrasonography revealed a 15 cm right ovarian solid mass. The patient underwent an exploratory laparotomy. The intraoperative findings were unremarkable except for the right ovarian mass. Peritoneal washing fluid was collected for cytology. A right salpingooophorectomy, right pelvic and para-aortic lymph node and omental biopsies, and appendectomy were performed. The initial diagnosis of the right ovarian mass was FIGO stage IA endometrioid adenocarcinoma grade 2. Postoperatively, the patient was referred to Chiang Mai University Hospital for further management. The review of the histologic slides of the initial tissue samples raised a differential diagnosis of Sertoli cell tumor. Immunohistochemical staining was performed and the residual gross specimen was reviewed with additional tissue sampling. The pathological diagnosis was reported as proliferative struma ovarii with atypical features, and a clinical metastatic work-up was recommended.

The patient underwent a thyroid scan which showed no evidence of a primary thyroid lesion. No clinical evidence of a metastatic lesion was detected in the chest X-ray and abdominal computed tomography (CT) scan. Close surveillance was planned, but the patient was lost to follow-up. Fifteen months after surgery, she presented with a dry cough for 1 month. A chest X-ray and CT scan revealed multiple and bilateral pulmonary nodules, consistent with metastatic lesions. No intra-abdominal disease was identified in the CT scan. The serum thyroglobulin level was 247.3 ng/mL (normal range ≤ 40 ng/mL). Recurrence of malignant struma ovarii was clinically considered. A pulmonary biopsy was not performed. The patient underwent a total thyroidectomy, followed by high dose radioactive iodine (I-131) therapy. The resected thyroid specimen was unremarkable. The clinical condition of the patient was stable and there was no progression of the pulmonary nodules seen after 3 episodes of iodine treatment during a 2-year period.

Forty-two months after the surgery, the patient developed hemoptysis and dyspnea. Chemotherapy (cisplatin and Adriamycin) was started and continued for 6 cycles. Five years after surgery, there was a progressive enlargement of the pulmonary nodules, accompanied by weakness of her left arm due to intramedullary spinal cord metastasis. The patient died of the disease 7 years after the initial surgery.

### 2.2. Pathological Findings

**Figure 1:**
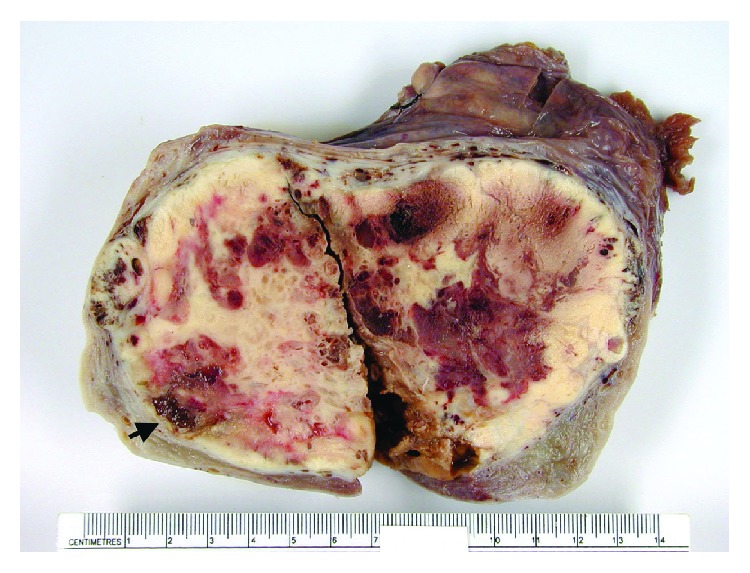
The right ovarian mass shows a solid yellow white sectioned surface with areas of necrosis and hemorrhage. A small gelatinous focus is observed (arrow).

Macroscopically, the right ovarian mass was a solid yellow white mass with areas of necrosis and hemorrhage (Figure 1). The capsule was intact. The review of the residual gross specimen with additional serial sectioning revealed few gelatinous tan foci and a 1 cm focus of gray sebaceous material.

**Figure 2:**
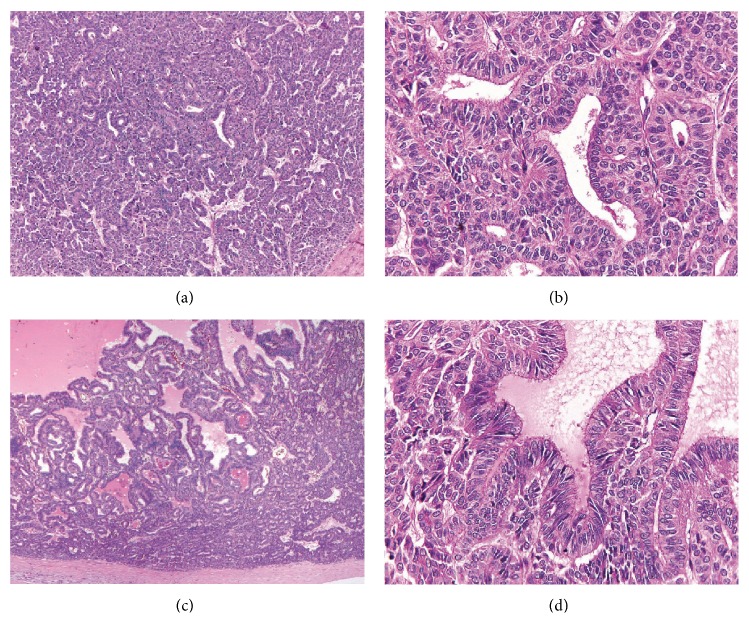
A significant histological appearance of the ovarian tumor. ((a) and (b)) Tubular pattern composed of columnar cells with uniform nuclei. ((c) and (d)) Focal endometrioid-like pattern characterized by dilated gland-like structures with papillary-like infolding.

**Figure 3:**
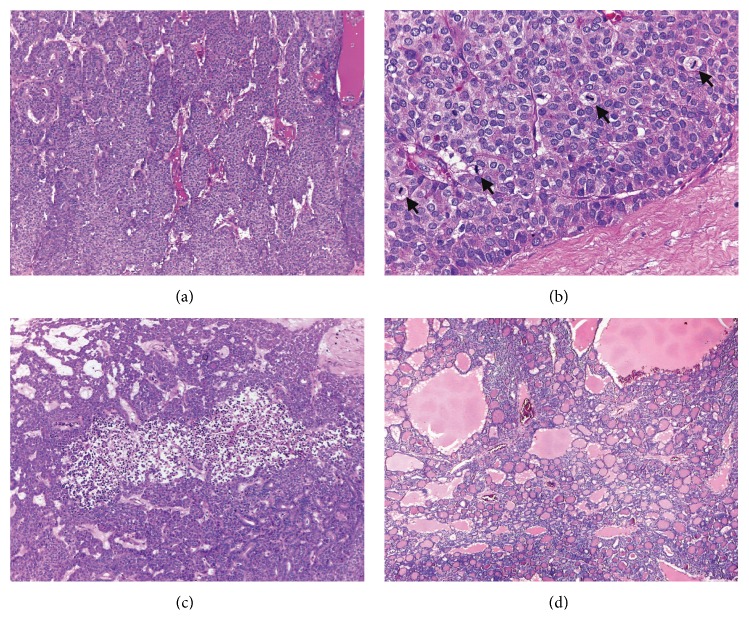
A poorly differentiated thyroid carcinoma component ((a) to (c)) and a typical thyroid area (d) in the ovarian tumor. ((a) and (b)) Insular and solid patterns composed of uniform cells with increased mitotic activity (arrows). (c) A focus of coagulative tumor cell necrosis. (d) Typical thyroid follicles with abundant colloid are identified in additional tissue samples.

**Figure 4:**
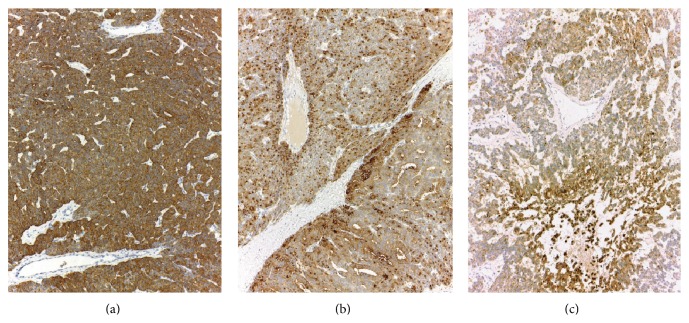
Positive immunohistochemical staining of ovarian tumor. (a) Cytokeratin 7. (b) Thyroglobulin (including an area of insular pattern in the top). (c) Epithelial membrane antigen.

Microscopically, the ovarian mass was composed of relatively uniform cuboidal to columnar cells with round to oval nuclei, predominantly arranged in a tubular pattern (Figures 2(a) and 2(b)). Dilated gland-like spaces with papillary-like infolding lined by elongated columnar cells simulating endometrioid adenocarcinoma were focally observed (Figures 2(c) and 2(d)). Solid tubules, a trabecular arrangement, and solid cell nests or an insular pattern were also present. The insular pattern (Figure 3(a)) accounted for approximately 20% of the tumor tissue. Mitotic activity was variable, ranging from absent or rare in the tubular areas to 11 in 10 high-power fields in the solid areas (Figure 3(b)). Large zones of previous infarction with hyalinized fibrosis were observed in the macroscopically necrotic areas. However, small foci of coagulative tumor cell necrosis were present within the neoplastic cell sheets (Figure 3(c)). Neither true papillary structures with fibrovascular cores nor ground glass/grooved nuclei were observed. Areas of typical thyroid-type tissue (Figure 3(d)) and a focus of mature cystic teratoma (dermoid cyst with cartilage) were identified in the second round of tissue sampling. Neither capsular involvement nor lymphovascular invasion was detected. Immunohistochemical stains showed a diffuse positive reaction for pancytokeratin (AE3/AE3), cytokeratin 7, and thyroglobulin (Figure 4), with focal reactions for epithelial membrane antigen (EMA) and vimentin. The immunostains for calretinin, CD99, chromogranin A, and synaptophysin were negative.

## 3. Discussion

Strumal lesions of the ovary may occasionally be confused with ovarian tumors, particularly when the typical thyroid or teratomatous component is not apparent [4, 5]. Foci of the usual-type teratomatous tissue may be small and sometimes cannot be histologically documented, even with extensive tissue sampling. A rare possibility of metastatic ovarian involvement from primary thyroid cancer should be borne in mind in the examination of an ovarian strumal lesion without an associated teratomatous component, and a correlation with the clinical data is required to rule out this possibility because the clinical history of primary thyroid cancer may be remote [8].

Histologically, the microfollicular pattern commonly present in ovarian strumal lesions tends to raise a differential diagnosis of granulosa cell tumor [2]. In the cases with tubular pattern, differential diagnoses may include Sertoli cell tumor and endometrioid adenocarcinoma (sertoliform variant). In addition, cord-like or trabecular arrangement and insular pattern may raise a concern of carcinoid tumor [4].

Sertoli cell tumor is an important differential diagnosis of an ovarian tumor with a tubular pattern in young women [5]. Sertoli cell tumor also shows a cord-like/trabecular arrangement, or a nest-like or solid pattern [5], which overlaps with the histological patterns in poorly differentiated thyroid carcinoma (i.e., trabecular, insular, or solid) as seen in this case. In addition, tumor cell necrosis and increased mitotic activity can be observed in Sertoli cell tumors [5].

Endometrioid adenocarcinoma may have a prominent tubular (sertoliform) pattern. A microglandular pattern in endometrioid adenocarcinoma may be similar to thyroid microfollicles. However, endometrioid tumors are uncommon in young women [9]. An association with endometriosis and/or adenofibroma and the presence of squamous differentiation are helpful features in supporting the diagnosis of an endometrioid tumor [9].

Carcinoid (or neuroendocrine) tumor is classified in the ovarian teratoma category and may occur in admixture with struma ovarii (strumal carcinoid) [1]. Carcinoid tumor may show insular and trabecular patterns and acinar/follicle-like arrangement which overlap with the features of poorly differentiated thyroid carcinoma. Although most ovarian carcinoid tumors have a favorable prognosis, the presence of increased mitotic activity and degree of nuclear atypia signifies the spectrum of atypical carcinoid tumor or neuroendocrine carcinoma which may exhibit aggressive behavior [10].

Immunohistochemical stains are helpful in confirmation of the diagnosis (Table 1). However, immunohistochemical stains can also be a pitfall when the strumal nature is not included in the consideration and the main differential diagnoses are only endometrioid tumor and Sertoli cell tumor. Using the immunohistochemical panel of EMA, CK7, inhibin, and calretinin, the EMA/CK7-positive and inhibin/calretinin-negative profile can lead to a misdiagnosis as an endometrioid tumor.

The diagnostic criteria for malignant struma ovarii were previously based on those applied to the identification of typical thyroid-type carcinomas. Due to the lack of a tumor capsule in struma ovarii, the capsular invasion criterion for thyroid follicular carcinoma was previously omitted in the evaluation of ovarian strumal follicular lesions [3]. Recently, extension of the tumor through the ovarian serosa forming a polypoid or nodular growth on the surface and/or the spread of tumor beyond the ovary at the diagnosis is considered to be a criterion for malignancy regardless of a bland-appearing or highly differentiated histomorphology of thyroid tissue [2, 11]. In addition to surface involvement or an extraovarian spread, a large tumor size (12 cm or more), dense surface adhesion, or ascites can provide a further clue regarding the risk of clinical disease progression [2, 7]. However, there are a minor proportion of cases in which the clinical detection of metastatic lesions during follow-up was the only indicator of malignant behavior [2].

Papillary carcinoma is the most common malignant form of struma ovarii and is promptly diagnosable by the presence of papillary architecture and well-recognized “ground glass” nuclei [2, 7]. Nevertheless, in the absence of true papillary structures, only the focal presence of ground glass nuclei is not sufficient for the diagnosis of papillary carcinoma in struma ovarii [2, 12]. For the diagnosis of follicular carcinoma arising in ovarian struma, vascular invasion is a useful feature but this is rarely observed [2, 7]. Thus, the presence of ovarian serosal invasion is an important diagnostic clue. Anaplastic carcinoma is recognized by marked nuclear atypia which should be distinguished from atypical nuclei in Hürthle cell change [2].

Poorly differentiated thyroid carcinoma represents a rare type of primary thyroid cancer and occurs extremely rarely in ovarian struma [7, 13]. Until recently, the diagnostic criteria of poorly differentiated thyroid carcinomas have been a controversial issue [14]. In recent consensus criteria, poorly differentiated thyroid carcinoma is characterized by a uniform population of small follicular cells arranged in a solid, trabecular, or insular pattern, commonly accompanied by significant mitotic activity (≥3 in 10 high-power fields), tumor necrosis, or convoluted nuclei, in the absence of typical nuclear characteristics of papillary carcinoma [14]. Using these criteria, the minor solid component with increased mitotic activity and focal necrosis in the present case (Figure 3) is qualified for a diagnosis of poorly differentiated thyroid carcinoma arising in struma ovarii. The presence of a poorly differentiated carcinoma component is an adverse prognostic indicator as observed in thyroid cancer. Even a minor poorly differentiated component (10–50%) within usual follicular thyroid carcinoma is associated with a significantly decreased survival [15].

The clinical course of thyroid carcinoma arising in struma ovarii is typically protracted with late recurrence and long survival duration in most cases [2]. In patients with malignant histological features, the 10-year and 25-year survival rates were 81% and 60%, respectively [2]. Among patients who died of disease, the median survival duration was 21 years in those with stage 1A, which was much longer than that of higher stage patients (5 years) [12]. In recent large studies on malignant struma ovarii, the clinical behavior was difficult to predict based on tumor histomorphology alone [2, 12, 16]. However, the presence of a minor component of anaplastic or poorly differentiated carcinoma appears to signify a poor clinical outcome. Patients with anaplastic or poorly differentiated thyroid carcinoma in struma ovarii had a shorter survival duration than those with the differentiated-type (papillary and follicular) carcinoma [2, 7].

Of 4 reported cases of poorly differentiated thyroid carcinoma arising in struma ovarii (including the present case), 3 patients were less than 30 years (22, 24, and 27 years) and at FIGO stage I (2 in stage IA and 1 in stage IC), whereas the remaining patient was 70 years of age and at stage II [7, 13, 17]. In 2 patients with available follow-up data, both received radioactive iodine treatment; the stage II patient died of the disease 3 years after the initial diagnosis [13], while the stage IA patient (the present case) died 7 years after the diagnosis.

In patients with advanced stage thyroid-type carcinoma arising in ovarian struma or those with tumor recurrence, thyroid ablation by surgery and radioactive iodine therapy has been recommended [6, 12]. The treatment in a stage I patient should be determined on an individual basis, with consideration of the risk and benefit to each patient [16]. Due to the poor prognosis of cases with anaplastic or poorly differentiated thyroid carcinoma in struma ovarii, stage IA patients with such component may benefit from an aggressive treatment approach [16].

## Figures and Tables

**Table 1:** Comparison of immunohistochemical stain results in the differential diagnoses.

	Ovarian strumal lesions	Endometrioid tumor	Sertoli cell tumor	Carcinoid tumor
Cytokeratin 7	+	+	−	+ or −
Epithelial membrane antigen	+	+	−	+
Calretinin/inhibin	−	−	+	−
Thyroglobulin/TTF-1	+	−	−	−
Chromogranin/synaptophysin	−	−	−	+
